# Environmental cues shape extracellular vesicles biogenesis and function in *Streptococcus pneumoniae*

**DOI:** 10.1186/s12866-026-04862-7

**Published:** 2026-03-04

**Authors:** Miriana Battista, Yann Bachelot, Teresa Franke, Christoph Saffer, Lioba Zimmermann, Laura Teuber, Marc Thilo Figge, Cláudia Vilhena

**Affiliations:** 1 Department of Biology, Bacterial Interface Dynamics lab, Chair of Pharmaceutical Biology, Friedrich-Alexander University Erlangen Nürnberg, Staudtstraße 5, Erlangen, 91058 Germany; 2 Department of Applied Systems Biology, HKI-Center for Systems Biology of Infection, Leibniz-Institute for Natural Product Research and Infection Biology, Jena, Germany; 3 Friedrich-Schiller-University, Jena, Germany; 4 Department of Infection Biology, Leibniz Institute for Natural Product Research and Infection Biology, Jena, Germany; 5 Friedrich-Schiller-University, Institute of Microbiology, Jena, Germany

**Keywords:** Metabolism, Biofilm, Carbon source, Vesicles, Biogenesis, Intercellular communication

## Abstract

**Background:**

The Gram-positive human pathogen *Streptococcus pneumoniae* adapts its metabolism to the environment during colonization and host invasion. Extracellular vesicles (EVs) are produced by *S. pneumoniae* in the process of infection but the exact interplay between metabolic adaptation and vesicle formation remains elusive. This study investigates the role of environmental cues in modulating pneumococcal EVs biogenesis and function.

**Results:**

Here, we demonstrate that exposure to normal human serum induced rearrangement of the pneumococcal cell wall and considerably increased Sp-EVs production. Temperature and pH were critical factors for Sp-EVs formation: 37 °C supported optimal EV production, while bacterial exposure to either basic or acidic environments slowed down pneumococcal EV biogenesis and led to a heterogeneous subpopulation profile. Proteomic analysis revealed that Sp-EVs are enriched in carbon metabolism-related proteins, specifically those associated with glycolysis (e.g. Eno, GapA, GapN, GpmA, PfkA, PykF, and Tpi). Moderate glucose availability enhanced Sp-EVs production and intracellular ATP level, underlying a relation between metabolic status and EV biogenesis. Functionally, Sp-EVs promoted biofilm formation in both *S. pneumoniae* and *Streptococcus pyogenes*. Sp-EVs isolated under glucose-rich conditions enhanced *S. pneumoniae* biofilms, whereas Sp-EVs from glucose-poor conditions strongly stimulated *S. pyogenes* biofilm formation.

**Conclusions:**

These findings underscore the role of host and environmental cues in shaping pneumococcal EV production, composition, and function, highlighting their potential involvement in metabolic adaptation and interspecies interactions.

**Supplementary Information:**

The online version contains supplementary material available at 10.1186/s12866-026-04862-7.

## Introduction

Bacteria constantly adapt to changing environmental conditions in order to survive and propagate [1, 2]. A set of metabolic, genetic and general regulatory rearrangements take place, shaping the course of bacterial evolution [3–6]. For instance, bacteria can evolve to acquire antibiotic resistance [7] or to specialize in the consumption of a specific source [8]. Environmental factors such as temperature, pH, osmolarity, and nutrient availability influence bacterial physiology [9–12]. When in contact with the human host, bacteria experience a wide array of stimuli which are sensed by surface exposed receptors and can trigger stress responses that impact bacterial fitness and hence survival [12–14]. Such stimuli range from immune molecules, antimicrobial peptides, cellular surfaces, hormones, reactive oxygen species, etc [15, 16].

*Streptococcus pneumoniae* is a Gram-positive human pathogen and the causal agent of complicated diseases as community-acquired pneumonia, meningitis and sepsis [17, 18]. In *S. pneumoniae*, metabolic adaptation plays a key role in colonization and pathogenicity. For instance, during colonization of the nasopharynx, glucose and hyaluronic acid sources are prioritized [19, 20] and endogenous hydrogen peroxide (H_2_O_2_) is produced via SpxB, acting as a signal to activate oxidative stress defense pathways [21]. Another event that occurs in diverse biological contexts, including changing environmental conditions and/or host exposure is the production of extracellular vesicles (EVs) [22].

EVs are non-replicative membrane-enclosed structures, ranging in size from 30 to 300 nm and originate from all kingdoms of life including microbial organisms [22–25]. They contain proteins, lipids, RNA and DNA and play relevant physiological roles such as waste disposal, membrane remodeling and cell-to-cell communication [26, 27]. Besides their physiological role, bacterial EVs can be implicated in, e.g. biofilm formation, cellular damage and immune modulation of the host [28–31]. Specifically, *S. pneumoniae* EVs (Sp-EVs) have been shown to modulate host cytokine and chemokine expression and release despite their non-cytotoxic feature [32–37]. Moreover, Sp-EVs downregulate expression of an endothelial surface marker and are internalized by human endothelial cells [37]. In our previous work, we utilized a co-cultivation (bacterial-human) multi-well model to investigate whether different stimuli, e.g. host-released substances, influenced vesicle synthesis [37]. We observed a co-cultivation time and concentration-dependent EV formation which suggests that host and pneumococcal EVs biogenesis are not independent mechanisms but appear to mutually influence each other in a highly dynamic and multifaceted manner.

Despite advances in understanding the environmental and metabolic regulation of bacterial pathogenesis, the interplay between EV formation, bacterial metabolism, and their functional roles in infection remains largely unexplored. How metabolic shifts and environmental signals shape EV biogenesis, and how this, in turn, influences bacterial survival, biofilm formation, and inter-species communication, remains an open question.

Here, we further characterized the role of human serum, temperature and pH on Sp-EVs biogenesis. We found glycolysis and general carbon metabolism pathways enriched in EVs proteome. A deeper dissection of glucose influence on Sp-EVs revealed a correlation between sugar abundance and EVs concentration which relates to cellular ATP content. Finally, we tested the functional role of glucose-derived EVs and conclude that Sp-EVs formed under high sugar abundance led to distinct bacterial growth phenotypes and distinct intra- and inter-species biofilm formation.

## Material and methods

### Bacterial strains and growth conditions

Two bacterial strains were used in this study: *Streptococcus pneumoniae* D39 and *Streptococcus pyogenes* M1 [38, 39]. Strains were grown in liquid Todd- Hewitt broth (Roth^®^) supplemented with yeast extract (THY) at 37 °C with 5% (vol/vol) CO_2_. Blood agar plates were prepared from Blood agar (VWR^®^) with addition of 5% (vol/vol) defibrinated sheep blood (Thermo Scientific^®^). Growth was monitored by measuring the optical density at 600 nm (OD_600_).

### Super- Resolution Structured Illumination Microscopy (SR-SIM) for bacteria interaction with Normal Human Serum (NHS)

For interaction of *S. pneumoniae* with normal human serum (NHS), human blood was obtained from the accredited Transfusion Medicine Unit, University Hospital Jena, Germany, Red Cross (accreditation D-ML-13153-01-01, EFI standards, DIN EN ISO 15189), with institutional approval and donor informed consent in accordance with ethical guidelines. Blood was taken from three healthy voluntary participants (who were not vaccinated against *S. pneumoniae*) in BD Vacutainer^®^ Clot Activator Tubes (BD Biosciences).

After 15 min of incubation, the blood was centrifuged at 4,000 x g for 15 min at room temperature (RT). The serum (referred to as normal human serum, NHS) was separated from the clot, kept at -20 °C and defrosted only shortly before experiments.

To study interaction of *S. pneumoniae* with NHS, Super- Resolution Structured Illumination Microscopy (SR-SIM) was performed. *S. pneumoniae* D39 was grown in suspension (≈ 10^8^ CFUs) and pelleted at 7,200 x g for 3 min at 4 °C. The bacterial pellet was resuspended in 500 µL of serum and incubated for 30 min at 37 °C and 5% CO_2_. After incubation, pneumococci were separated from the serum by centrifugation at 7,200 x g for 3 min at 4 °C.

Bacteria were stained with a mixture of 4’,6-Diamidino-2-Phenylindole, dihydrochloride (DAPI) (1:500) and wheat germ agglutinin (WGA) CF^®^488 (1:1000) in phosphate buffered saline-Tween (PBST) for 1 h at RT. Bacterial cells were then washed and fixed with 4% (vol/vol) paraformaldehyde (PFA) solution for 15 min at 4 °C.

For the SR-SIM imaging, 10 µL of the sample was spotted on 1% (vol/vol) agarose pads. Agarose pads were covered with No. 1.5 coverslips (Roth^®^) and stored at 4 °C for further imaging. The SR-SIM data were acquired on an Elyra 7 system (Zeiss) equipped with a 63×/1.4 NA Plan-Apochromat oil-immersion DIC M27 objective lens (Zeiss), a Piezo stage, and a PCO edge sCMOS camera with 82% QE and a liquid cooling system with 16-bit dynamic range. Using Lattice SIM mode, images were acquired with 13 phases. WGA CF^®^ 488 was detected with a 488-nm laser and a BP 495–590 emission filter and DAPI was detected with a 405-nm laser and a 477/35 emission filter. Super resolution images were computationally reconstructed from the raw data sets using default settings on ZenBlack software (Zeiss) Images were analyzed using the Fiji ImageJ software [40].

### Extracellular vesicles isolation

For bacterial extracellular vesicles (EVs) isolation, *S. pneumoniae* D39 strain was grown on a solid blood agar plate overnight, and single colonies were inoculated into THY and grown at 37 °C with 5% (vol/vol) CO_2_. To test various environmental conditions, *S. pneumoniae* was alternatively grown at 30° C, at different pH conditions (pH 5 and 8) and in presence of sugar or NHS. For this, THY was further supplemented with acetic acid (0.6 g/mol) (Sigma-Aldrich^®^) or sodium hydroxide (0.4 g/mol) (Sigma-Aldrich^®^), D-(+)-glucose (10, 50, 100 and 200 mM) (Merck^®^) or NHS. At mid-logarithmic growth phase, 50 mL aliquots of bacterial culture were centrifuged twice (10,000 x g, 20 min, 4 °C and 4,000 x g, 20 min, 4° C). The pellet was discarded, and the supernatant was filtered through a 0.45-µm pore membrane (Sartorius^®^) in order to obtain cell-free supernatant. The resulting cell-free media was ultracentrifuged at 100,000 x g for 2 h at 4 °C (Rotor SW 45 Ti, Optima XPN-80 Ultracentrifuge, Beckman Coulter, Life Sciences). The resulting vesicle pellet was resuspended in 1 mL sterile Milli-Q water and stored at 4 °C for further analysis. For the environmental influence analysis, EVs were alternatively precipitated using ExoQuick-TC (System Biosciences) according to the manufacturer’s protocol. For subsequent use, EVs were stored at -80 °C and thawed when needed.

### Extracellular vesicles counting

To determine size and concentration of isolated vesicles, nanoparticle tracking analysis was performed using the ZetaView^®^ (Particle Metrix GmbH) with a detection wavelength at 488-nm in scatter mode equipped with ZetaView software (version 8.05.16 SP3). Isolated vesicles were dispersed in 1 mL of Milli-Q water and diluted suitably.

For the experiments testing the environmental influence, isolated vesicles were counted using an NS300 dynamic light-scattering microscope (Malvern Panalytical) fitted with NanoSight NTA 3.2 software. Videos were captured at 24 fps for three periods of 60 s for each sample and analyzed using NanoSight NTA 3.2.

### Protein-protein interaction network analysis

A protein-protein interaction (PPI) network was constructed based on previously acquired proteomic data, which can be accessed online in this reference [37], of THY Sp-EVs, to identify key functional relationships among differentially expressed proteins. First, proteins identified from *S. pneumoniae* D39 strain were mapped to the well-annotated R6 reference strain identifiers using BLASTp [41] with default parameters. A summary of statistics of the mapping is provided in Supplementary Table 1 (Table S1). Subsequently, the mapped R6 protein identifiers were used to construct the protein interaction network using the STRING database [42]. Protein interactions were inferred based on integrated evidence from multiple sources, including experimentally validated interactions, curated databases, co-expression data, genomic context predictions, and text mining, with a confidence threshold of 0.7. Network visualization was performed using Cytoscape [43]. Functional enrichment analysis of the network was conducted using KEGG pathway annotations to reveal biological processes and pathways associated with the identified protein interactions. Over-representation was evaluated using hypergeometric tests and Benjamini–Hochberg correction for multiple comparisons. Pathways with an adjusted p-value ≤ 0.05 were considered significantly enriched.

### ATP measurement

To quantify ATP in bacterial cells grown in the presence of sugar abundance, *S. pneumoniae* D39 was grown in THY with or without D-(+)-glucose (10, 50, 100 and 200 mM) (Roth^®^) at 37 °C with 5% (vol/vol) CO^2^ until mid-logarithmic growth phase was reached. Bacteria were seeded in a 96-well plate and the BacTiter-Glo™ Microbial Cell Viability Assay was performed according to the manufacturer’s protocol. In this assay, luminescence is the result of mono-oxygenation of luciferin catalyzed by luciferase in the presence of Mg2^+^, ATP and molecular oxygen. The measured luminescent signal is proportional to the amount of ATP present and extracted from bacterial cells. The detected luminescent signal was normalized to the OD_600_ of the bacterial culture measured before the ATP extraction.

### Biofilm quantification

For quantification of biofilm formation, the Microtiter Dish Biofilm Formation Assay was performed with small changes [44]. Bacteria (OD_600_ 0,3) were incubated with pneumococcal EVs at two different concentrations (C1 and C2) and statically grown on 96-well plates to obtain biofilms (Thermo Scientific), for 18 h at 37 °C with 5% (vol/vol) CO_2_. Four different EVs conditions were tested: EVs isolated from *S. pneumoniae* D39 culture in THY with or without D-(+)-glucose (10, 50, and 200 mM). After 18 h, the supernatant was transferred to another plate and OD_600_ measured as a read for planktonic growth. To each well of the original plate, 200 µL of a 1% (vol/vol) crystal violet solution was added and the plate was incubated for 30 min at room temperature. After repeated washing steps, the plate was left to dry for 1 h. Ethanol [95% (vol/vol)] was added to each well and left for 30 min at room temperature. The released crystal violet was finally transferred to a new 96-well plate, and absorbance at 620 nm was measured.

### Spot assay

Bacterial cells were incubated in a 96-well plate with pneumococcal EVs at 37 °C with 5% (vol/vol) CO_2_ for 18 h to test biofilm formation as previously described. After 18 h, planktonic cells suspension was transferred to another plate and OD_600_ was measured. For spot assay, planktonic cells were serially diluted, spotted onto blood agar plates and grown at 37 °C and 5% (vol/vol) CO_2_ overnight. Four different EVs conditions were tested: EVs isolated from *S. pneumoniae* D39 culture in THY with or without D-(+)-glucose (10, 50, and 200 mM).

### Statistical analysis

Unless otherwise stated, statistical significance was determined by ordinary one-way analysis of variance (ANOVA) test with a Tukey multiple comparisons test. Probability values (p-values) were defined as follows: ns, *p* ≥ 0.1; *, *p* ≤ 0.05; **, *p* ≤ 0.01; ***, *p* ≤ 0.001; ****, *p* ≤ 0.0001. In figures, asterisks denote statistically significant differences, whereas the absence of asterisks indicates non-significant comparisons. Statistical analysis was performed using Prism version 10 for Windows (GraphPad Software, La Jolla, CA, USA).

## Results

### Pneumococcal cell wall arrangement and Sp-EVs formation are affected by human serum

**Fig. 1:**
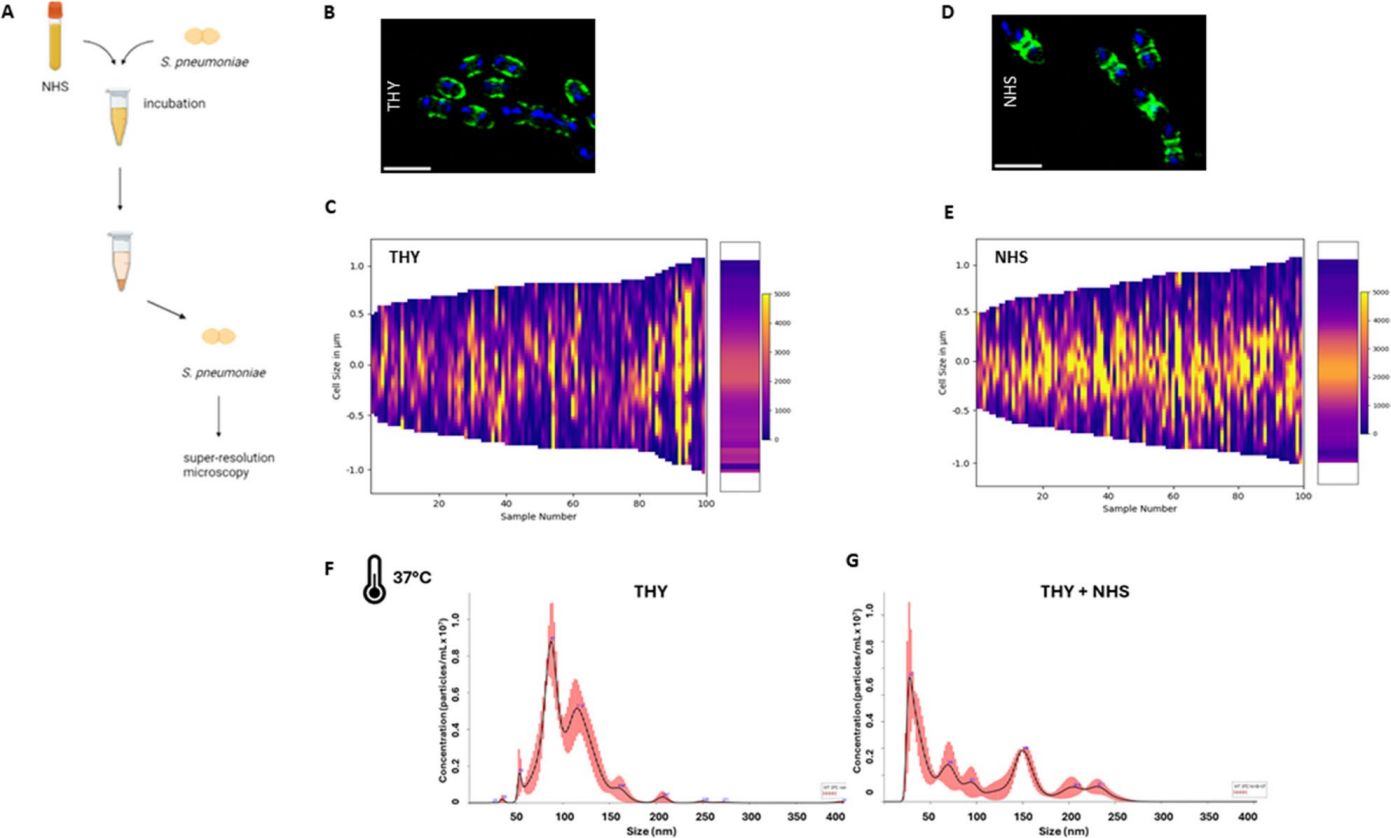
Effect of normal human serum on *S. pneumoniae* D39 cell wall and Sp-EV production. **A** Schematic representation of the experiment performed to investigate the interaction of *S. pneumoniae* D39 with normal human serum (NHS). Mid-exponentially grown D39 bacterial cells were incubated with NHS for 30 min, centrifuged, stained and visualized by super-resolution structured illumination microscopy (SR-SIM). Representative SR-SIM images of pneumococci stained with WGA CF^®^488 (green) and DAPI (blue) in (**B**) growth medium (THY, control condition) and (**D**) after incubation with NHS. Scale bar = 2 μm (**C**,** E**) Graphs showing distribution of WGA across the bacterial cell wall in the control condition (THY) and in NHS-challenged bacterial cells, respectively. Fluorescence intensity range from 0 (dark blue/purple) to 5000 (yellow) arbitrary units. **F**,** G** Histograms representing Sp-EVs concentration (particles/mL × 10^7^) and size (nm), after three rounds of nanoparticle tracking analysis (NTA). EVs were isolated from supernatant of *S. pneumoniae* D39 grown until mid-logarithmic phase in THY at 37 °C with or without NHS. Red lines represent standard deviation (*n* = 3)

Bacteria cell wall composition and EVs production can be influenced by different environmental conditions and host-derived factors such as serum components [45, 46]. Thus, we investigated the interaction of *S. pneumoniae* D39 with normal human serum (NHS) and visualized bacterial cell morphology by super-resolution microscopy (Fig. 1A). Mid-exponential phase D39 cells were incubated with NHS and later stained with fluorescently labeled wheat germ agglutinin (WGA), which is a carbohydrate-binding lectin with high affinity for N-acetylglucosamine (a main structural component of the pneumococcal cell wall), and DAPI for DNA.

The distribution of WGA across the bacterial cell wall in the control condition (cells growing in THY) was random and dispersed across the entire wall, as seen in the representative microscopy picture and on the distribution graph (Fig. 1B and C). A certain degree of heterogeneity among single individual bacteria could be observed, however without a correlation between cell length and signal intensity. For bacterial cells challenged with NHS, the signal distribution was different, and WGA appeared mainly localized at mid-cell, suggesting a cell wall rearrangement compared to the control (Fig. 1D). Quantification of signal intensity corroborated the findings, revealing a higher accumulation of WGA at the septum and flanking-septum regions in bacterial cells under NHS challenge (Fig. 1E).

In a previous work, we have documented the effect of co-cultivating human endothelial and bacterial cells on pneumococcal EVs (Sp-EVs) formation [37]. Given the effect of NHS on the bacterial cell wall arrangement, we wondered if NHS would affect EV formation.

To ascertain the influence of NHS on EVs formation, pneumococci were grown in the absence and presence of NHS at 37 °C, EVs were isolated and further characterized by nanoparticle tracking analysis (NTA). Media controls (without bacteria) were performed and showed significantly less EVs than the conditions with bacteria (Fig. S1). Histograms representing particles profile showed a different size distribution and concentration of EVs isolated from NHS-challenged bacteria compared to the control (Fig. 1F and G). EV population of the control condition depicted a typical size distribution, with one main population around 80–100 nm (Fig. 1F). Under NHS challenge, the EVs populations appeared more heterogeneous, with several subpopulations emerging in the size range 50–150 nm (Fig. 1G).

Taken together, these results suggest a different cell wall arrangement of *S. pneumoniae* D39 and a modulation of EV formation when encountering human serum factors.

### Sp-EVs production is sensitive to temperature and pH variation

We showed that pneumococcal EVs production was modulated by the presence of NHS. Building on this observation, we investigated the influence of additional environmental factors such as temperature and pH.

**Fig. 2:**
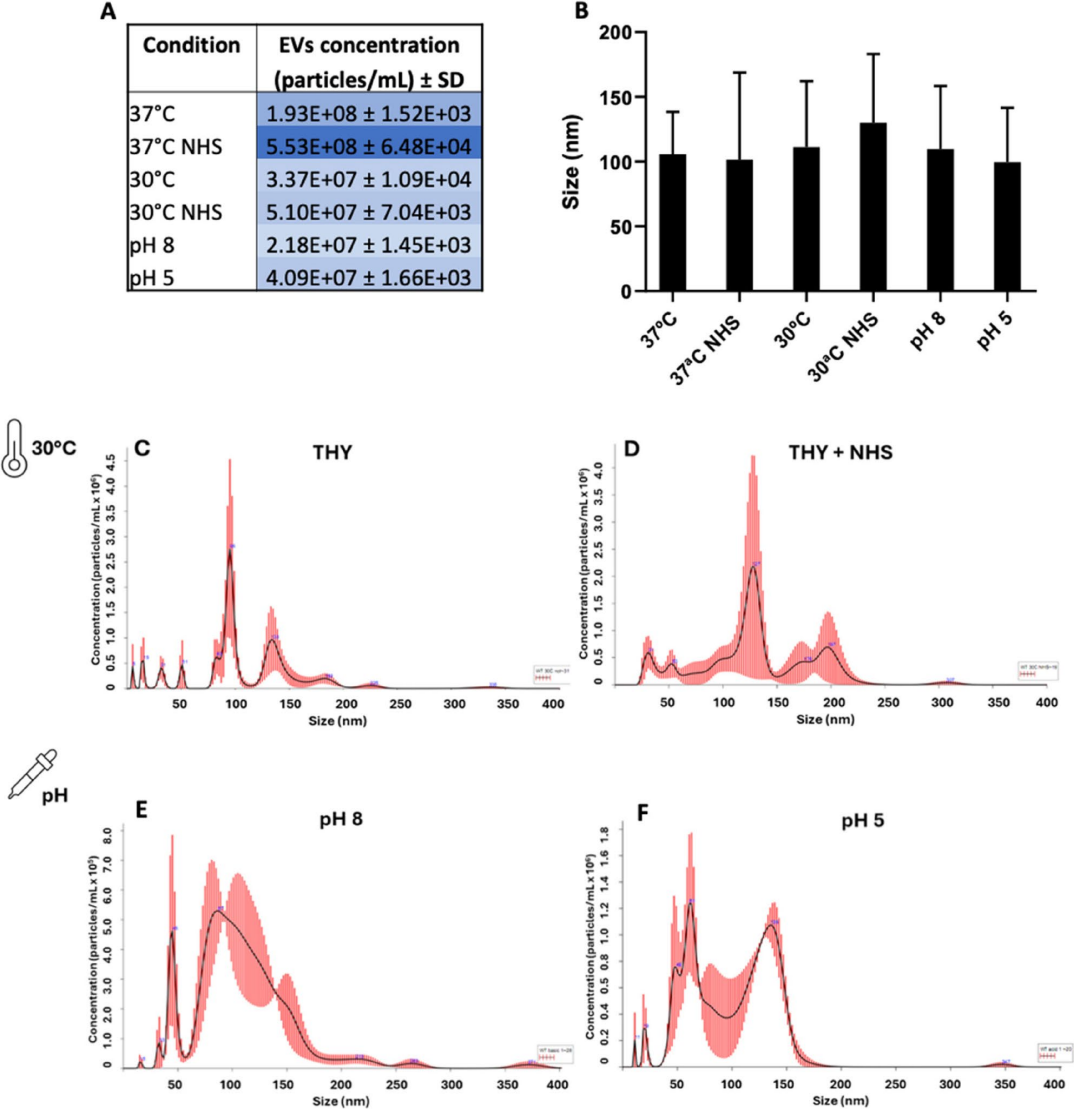
Characterization of Sp-EVs produced under varied environmental conditions. **A** Table representing Sp-EVs concentration (particles/mL) and (**B**) graph showing Sp-EVs size (nm) after nanoparticle tracking analysis. EVs were isolated from the supernatant of *S. pneumoniae* D39 grown until mid-logarithmic phase in THY at 37 °C–30 °C with or without NHS or at different pH conditions. Mean ± SD (*n* = 3). **C-F** Histograms showing particle profile for size (nm) and concentration (particle/mL) of EVs isolated from bacterial culture at 30 °C (**C**) in the absence or (**D**) in the presence of NHS and (**E-F**) at different pH conditions (pH 8 and 5). Red lines represent standard deviation

Consistently, our results showed that Sp-EVs concentration was severely affected by distinct environmental conditions (Fig. 2A) but not the average size of the population (Fig. 2B**)**. Sp-EVs production was stimulated by the presence of NHS, but temperature emerged as a critical factor for this process. When pneumococci were grown at 37 °C, Sp-EVs concentration in the bacterial supernatant was high (1.93 × 10^8^ particles/mL) and further increased under NHS condition (5.53 × 10^8^ particles/mL). In contrast, a temperature of 30 °C led to reduced EVs concentration despite the presence of NHS (5.10 × 10^7^ particles/mL) (Fig. 2A). A similar size profile was observed for Sp-EVs isolated from pneumococci grown at 30 °C with or without NHS (Fig. 2C and D).

Additionally, variations on pH drastically affected Sp-EVs production. EVs concentration decreased when pneumococci were exposed to either basic or acidic environment (pH 8 and pH 5) in comparison with the THY control (Fig. 2A). The number of subpopulations increased with the alkalinity of the media (Fig. 2E) which goes in line with the previously observed effect of aggregation of EVs at acidic pH, leading to a wider and single subpopulation [47].

In summary, our findings demonstrate that a temperature of 37 °C is the optimal condition for *S. pneumoniae* to produce EVs under the tested conditions. Exposure to either basic or acidic environments, as well as temperature shifts, negatively affects Sp-EVs production. In this context, environmental cues emerge as key aspects to consider in the study of Sp-EVs biogenesis.

### Carbon metabolism-related proteins are enriched in Sp-EVs cargo

We showed that Sp-EVs production is influenced by environmental factors such as the presence of human serum, temperature and pH variations. Bacterial EVs cargo is, in turn, dynamically adapted according to environmental conditions [48].

**Fig. 3:**
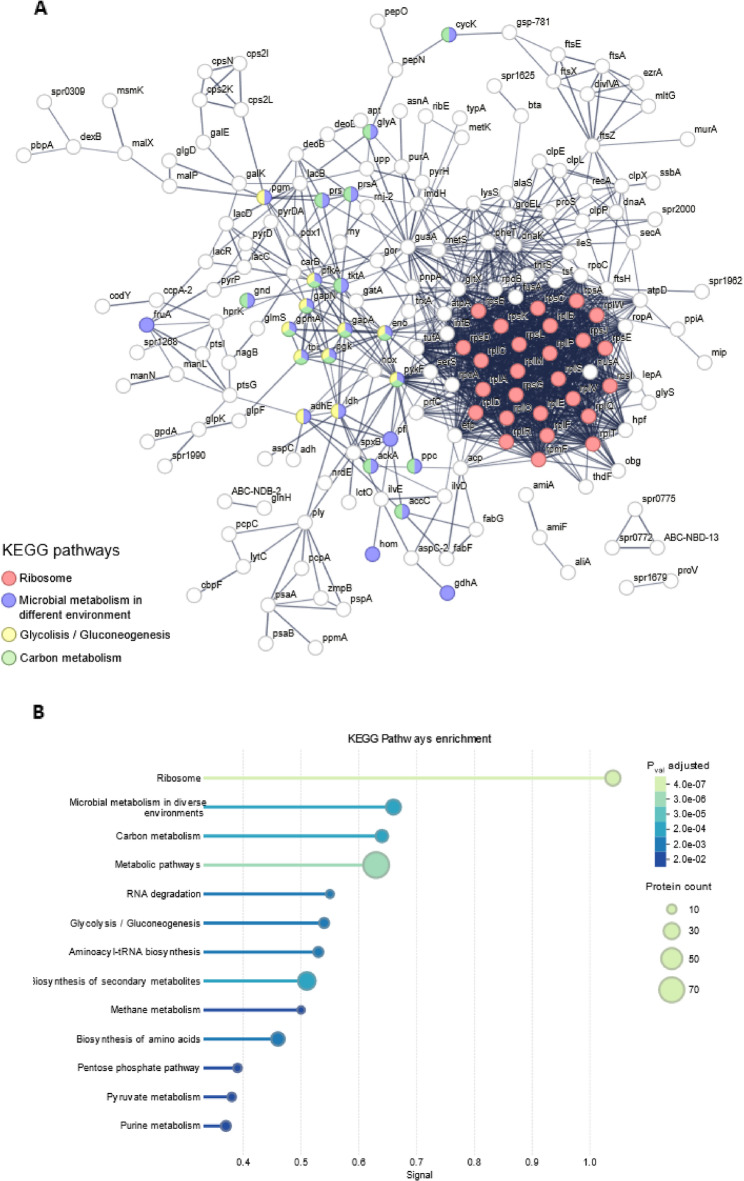
Protein-protein interaction network and enrichment analysis of Sp-EVs proteome. **A** STRING network of the proteins found in Sp-EVs (THY) in the proteomic analysis. Nodes and edges represent respectively the different proteins and the proteins’ interactions with high confidence (interaction score > 0.7). Proteins involved in pathways of interest are highlighted with different colors. **B** Functional enrichment of the network against the KEGG database. Horizontal bars show the –log10 of the adjusted p-value for every pathway that is significantly over-represented (*p* ≤ 0.05). Circle area is proportional to the number of Sp-EV proteins assigned to the corresponding pathway

In our previous work, proteomic profiling on EVs isolated from *S. pneumoniae* D39 and clinical isolated strains revealed a diverse protein composition, ranging from choline-binding proteins, cell division-related proteins, capsular polysaccharide biosynthesis, etc [37]. However, this analysis primarily focused on the differential protein abundance between strains (wild-type vs. clinical isolated) and did not address the functional relationships or potential co-associations between proteins. To bridge this gap and understand the functional significance and interaction networks of Sp-EVs proteins, we constructed a protein-protein interaction (PPI) map using STRING [49] (Fig. 3A).

Functional annotation through KEGG pathway enrichment showed significantly enriched pathways in which Sp-EVs proteins are involved. Among the enriched pathways with higher signal, we found *ribosome*, *microbial metabolism in diverse environments*, *carbon metabolism*, *metabolic pathways*, *RNA degradation* and *glycolysis/ gluconeogenesis* (Fig. 3B).

The presence of ribosome-related proteins in Sp-EVs is in accordance with previous observations for EVs isolated from other Gram-positive and Gram-negative bacteria [50, 51]. Interestingly, a substantial subset of Sp-EVs proteins mapped to core metabolic pathways, specifically to carbon metabolism and glycolysis or gluconeogenesis (e.g., Eno, GapA, GapN, GpmA, PfkA, PykF, and TpiA). Eno, GapA and TpiA are known to bind human plasmin and plasminogen and to contribute to invasion [52–54].

These findings suggest possible involvement of Sp-EVs in mechanisms of bacterial metabolic adaptation, potentially facilitating survival and persistence under changing environmental conditions.

### Sp-EVs production is promoted by. moderate glucose availability

PPI analyses of the proteins found in Sp-EVs cargo revealed enrichment in carbon metabolism-related proteins, several of which are involved in glycolysis or gluconeogenesis pathways. Glucose metabolism is known to regulate pneumococcal virulence properties such as capsule synthesis, rapid growth in the bloodstream, adherence and immune evasion in the nasopharynx [55–57]. Pneumococcal extracellular vesicles, in turn, play a role in infection mechanisms and have been shown to carry virulence factors [32, 35, 37]. Bringing these concepts together, we wondered if glucose availability would impact bacterial capacity to synthesize EVs.

To investigate the possible connection between glucose metabolism and Sp-EVs formation, pneumococci were grown in THY with increasing concentrations of glucose (0,10, 50, 100 and 200 mM) and Sp-EVs production was assessed.

**Fig. 4:**
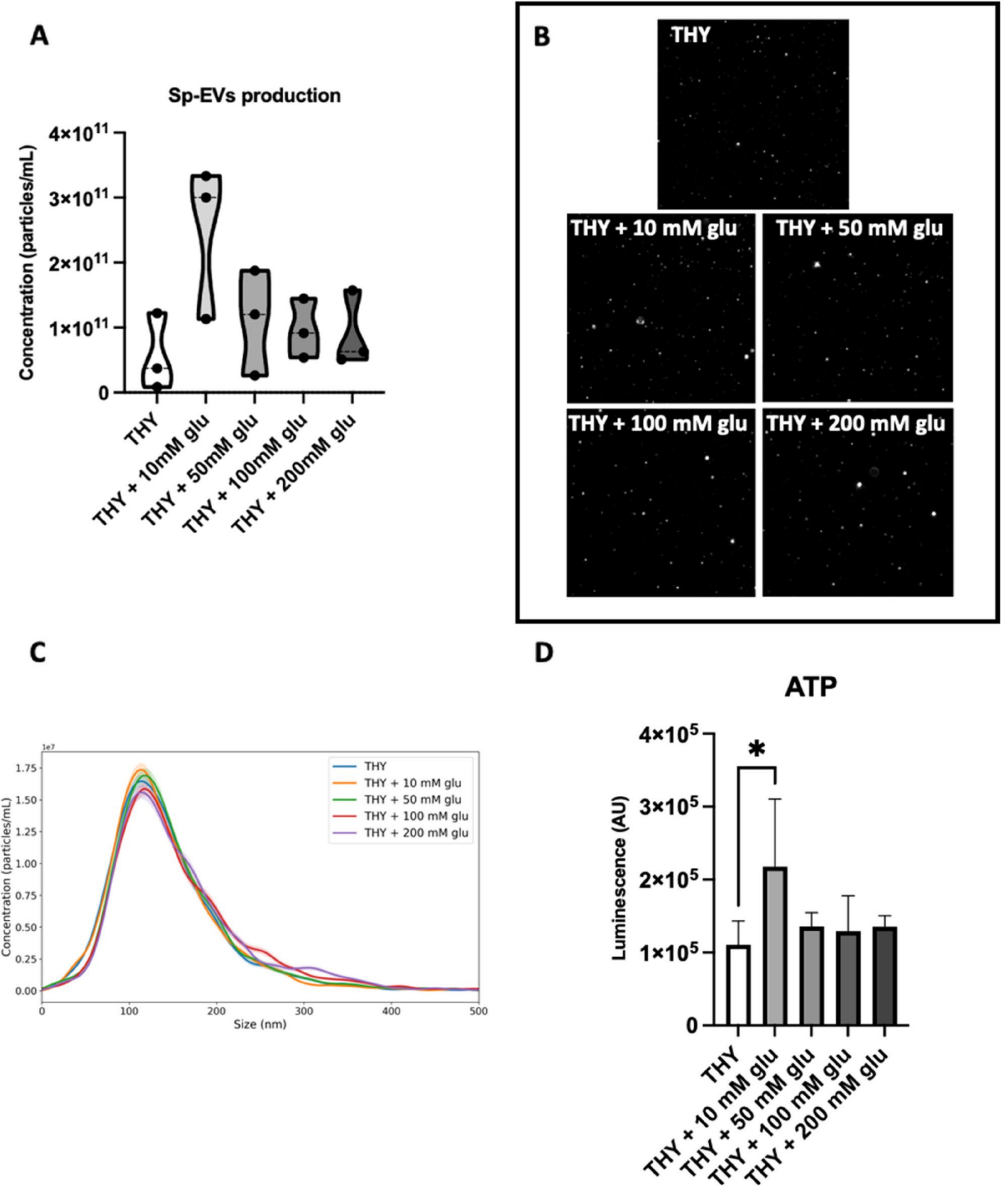
Glucose influence on Sp-EVs formation and ATP production by *S. pneumoniae* D39. **A** Violin plot representing Sp-EVs concentration (particles/mL) after nanoparticle tracking analysis. EVs were isolated from the supernatant of *S. pneumoniae* D39 grown until mid-logarithmic phase at 37 °C in THY with D-(+)-glucose (10, 50, 100 and 200 mM). A condition with *S. pneumoniae* D39 grown in THY without D-(+)-glucose was used as control. The detected Sp-EVs concentration was normalised to the bacterial culture OD_600_ measured before EV isolation. Individual points correspond to independent biological replicates (*n* = 3). The median is indicated by the central line, and the violin shape illustrates the distribution and batch-to-batch variability. Ordinary one-way ANOVA test with Tukey’s multiple comparisons test. **B** Representative snapshots of Sp-EVs, visualized by dynamic light-scattering microscopy. **C** Histogram showing particle profile for concentration (particles/mL) and size (nm) of Sp-EVs in all tested conditions. **D** Graph representing ATP production in *S. pneumoniae* D39 cells grown in THY with or without D-(+)-glucose (10, 50, 100 and 200 mM) at 37 °C until mid-logarithmic growth phase. The amount of ATP present and extracted from bacterial cells was measured as luminescent signal and expressed in AU. The detected luminescence signal was normalised to the OD_600_ of the bacterial culture measured before the ATP extraction. Mean ± SD (*n* = 3). One-way ANOVA followed by Fisher’s LSD post-hoc test

Sp-EVs were produced in more abundance when bacteria were exposed to a lower glucose concentration (10 mM), followed by a marked decrease in total vesicle amount with increasing glucose concentrations (50, 100, 200 mM) (Fig. 4A). This inverse relationship suggests that EV biogenesis may be sensitive to metabolic status. Representative light scattering microscopy images (Fig. 4B) and particle profile (Fig. 4C) of Sp-EVs isolated under glucose abundance showed a homogenous morphology and a unimodal sized-population.

Glucose metabolism is directly linked to ATP production in bacterial cells [20] and ATP levels are regulated according to cellular energy demands for key cellular processes [58]. Therefore, intracellular ATP level was measured after growing pneumococci in the presence of glucose (Fig. 4D). ATP amount was significantly higher in the 10 mM glucose condition compared to the control. The similar trend observed for Sp-EVs production and intracellular ATP level in response to environmental glucose availability suggests that a moderate glucose concentration (10 mM) is favorable for *S. pneumoniae* and may promote EV formation.

### Sp-EVs influence intra- and interspecies biofilm formation

So far, our results have shown that *S. pneumoniae* EVs production is influenced by environmental factors, including pH and glucose availability, suggesting that Sp-EVs may play a role in mechanisms of bacterial metabolic adaptation. Bacterial EVs are also known to contribute to biofilm formation, facilitating structural development, nutrient transport, and intercellular communication [30–60].

**Fig. 5:**
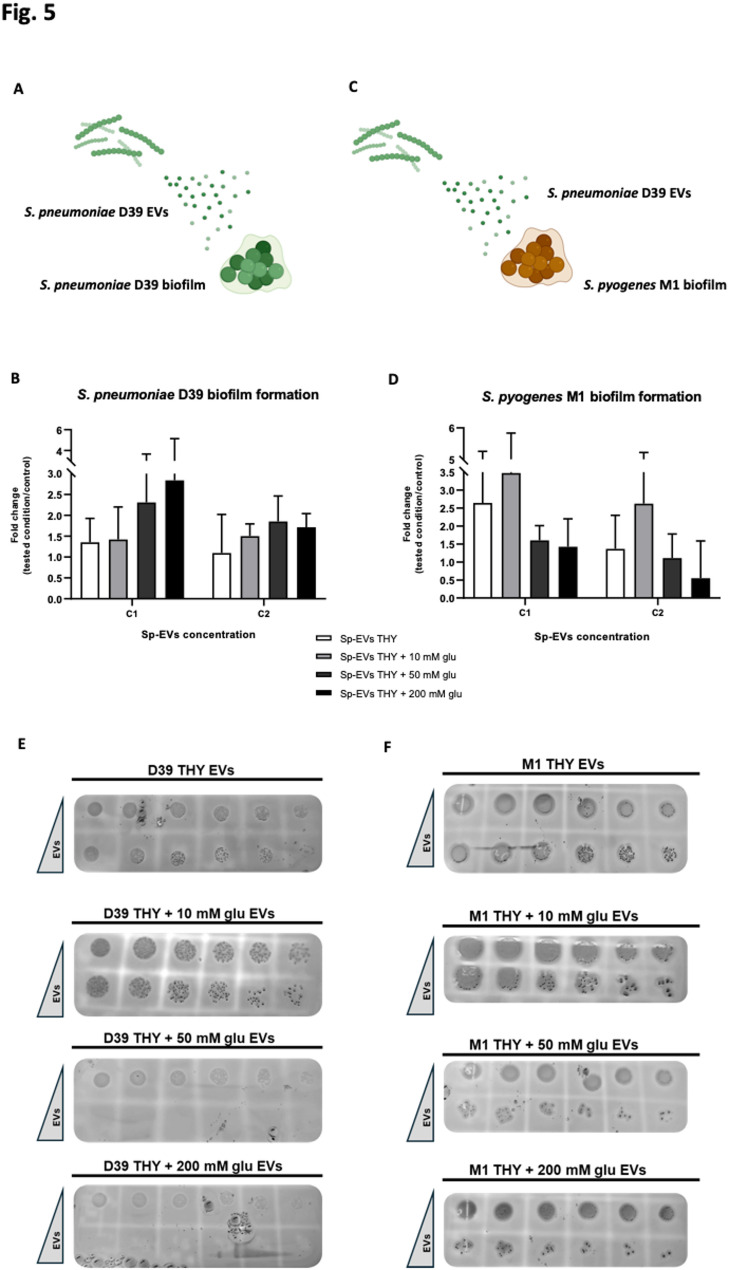
Sp-EVs influence on biofilm formation of *S. pneumoniae* D39 and *S. pyogenes* M1. **A**,** C** Schematic representation of EVs effect on biofilm formation from different species. Quantification of biofilm formation by (**B**) *S. pneumoniae* D39 or (**D**) *S. pyogenes* M1 after incubation for 18 h with Sp-EVs at two different concentrations (C1 = ~ 3 × 10^9^; C2 = ~ 4,8 × 10^10^). Sp-EVs were isolated from the supernatant of pneumococci grown until mid-exponential phase at 37 °C in THY with or without D-(+)-glucose (10, 50 and 200 mM) and were subsequently diluted in THY. A condition of biofilm formation by bacterial cells without Sp-EVs was used as control. Fold change (tested condition/control) ± SD (*n* = 3). Ordinary one-way ANOVA test with Tukey’s multiple comparisons test. **E**,** F** Representative images of spot assay performed to measure the viability of planktonic cells from the biofilm of *S. pneumoniae* D39 or *S. pyogenes* M1 after incubation with Sp-EVs for 18 h as previously described. D39 or M1 bacterial cultures were serially diluted (from left to right: 10^− 1^ to 10^− 6^), spotted onto blood agar plates and grown at 37 °C overnight to evaluate CFUs

To investigate the role of Sp-EVs in biofilm development and to further assess the potential impact of Sp-EVs produced under glucose abundance, biofilm formation of *S. pneumoniae* D39 in the presence of Sp-EVs was evaluated (Fig. 5A). Pneumococci were incubated with increasing concentrations of Sp-EVs (C1 or C2), isolated from mid-exponential bacterial cultures in THY with (10, 50, 200 mM) or without glucose.

Biofilm formation increased when pneumococci were grown for 18 h in the presence of Sp-EVs, compared to the control (bacteria grown in the absence of Sp-EVs) (Fig. 5B). Moreover, pneumococcal biofilm development was particularly triggered by the lowest concentration of Sp-EVs (C1), isolated under the highest glucose concentrations (50 and 200 mM).

Next, we investigated whether Sp-EVs could also influence biofilm formation of a different streptococcal species, *Streptococcus pyogenes* M1 (Fig. 5C), another Gram-positive human pathogen that colonizes the throat, nose and skin [61, 62]. We included *S. pyogenes* as a biologically relevant comparator to assess whether pneumococcal EV-mediated effects are species-specific and/or reflect conserved ecological responses within the shared respiratory niche. These pathogens share a niche and genus but differ in genomic regulation and capsule composition.

Similarly to the observed with the intra-species biofilms, Sp-EVs enhanced *S. pyogenes* M1 biofilm development, compared to the control without EVs (Fig. 5D). However, biofilm formation was tendentially higher when *S. pyogenes* M1 was exposed to Sp-EVs isolated from pneumococcal culture in THY without glucose or with a moderate glucose concentration (10mM). EVs derived only from THY medium supplemented with 200 mM glucose were also tested for their influence on biofilm formation of both species, to exclude a possible effect from non-bacterial EVs. We observed no increase in biofilm formation in this condition compared to the control condition (bacteria grown without EVs) (data not shown).

The viability of planktonic cells derived from the biofilm was also examined. Planktonic cell suspensions were serially diluted and spotted onto blood agar to determine the resulting colony forming units (CFUs). The viability of *S. pneumoniae* D39 planktonic cells decreased when pneumococci were incubated for 18 h with the higher Sp-EVs concentration, as shown by a reduction in CFUs. The decrease in planktonic cells viability was even more pronounced when Sp-EVs at the higher concentration were produced under increasing glucose concentrations (10, 50, 200 mM) (Fig. 5E). A similar outcome was observed for *S. pyogenes* M1 biofilm, where the reduction of planktonic cell viability was directly correlated to the Sp-EVs concentration and glucose abundance, although a greater number of CFUs was detectable compared to the *S. pneumoniae* D39 conditions (Fig. 5F).

Overall, our results suggest that Sp-EVs promote biofilm formation in both *S. pneumoniae* D39 and a different streptococcal species, *S. pyogenes* M1. Interestingly, EVs produced under glucose abundance, have opposing effects on intra- vs. inter-species biofilm formation.

## Discussion

The biogenesis and biological effects of extracellular vesicles (EVs) produced by the Gram-positive bacterium *Streptococcus pneumoniae* are still poorly understood. This study provides new insights into the environmental influence and functional roles of pneumococcal EVs (Sp-EVs), highlighting their potential role in bacterial metabolic adaptation and intra- or interspecies communication.

Bacteria constantly adapt to environmental conditions to ensure survival and proliferation [1, 2]. *S. pneumoniae* is a highly adapted human pathogen, capable of colonizing and invading its host, causing localized infections like otitis media and sinusitis, or more severe and life-threatening diseases like community-acquired pneumonia, bacteremia or meningitis [63]. The study of metabolic adaptation mechanisms in *S. pneumoniae* is essential for understanding its strategies for coping with the host environment. Bacterial regulatory systems are involved in sensing and responding to environmental signals, through the expression of virulence factors and proteins required to react to stressors such as antibiotics, temperature and pH variation and nutrient depletion [63].

We demonstrate that human serum impacts the pneumococcal cell wall structure and modulates Sp-EV formation (Fig. 1). This suggests that host-derived factors, such as human complement proteins, may induce remodeling of the bacterial cell wall, potentially facilitating EV release. Factor H, a human protein involved in complement regulation, protects the septum of the pneumococcus against C3 binding and consequently opsonization [64]. The septum is a highly dynamic subcellular location, where the cell wall is constantly being remodeled and thus a vulnerable place for host attack. In line with this, proteomic analysis revealed an abundance of septum-related proteins in Sp-EVs (e.g. DivIVA, EzrA, FtsA, FtsE, FtsX, FtsZ), suggesting a possible septal origin for EV formation (Fig. 3). Given the fact that WGA staining was enriched in the septal region and higher EV production occurred upon NHS incubation, we hypothesize that the membrane attack complex (MAC), also termed C5b-9, at the terminal complement cascade, might be inserted into the cell wall of bacteria, leading to pore formation and thus facilitating the observed higher release of EVs. We optimized a protocol for NHS-pneumococcus incubation and observed C5b-9 deposition on the bacterial surface on a time- and concentration-dependent manner (Fig. S2). Moreover, *S. pneumoniae*-derived serum (after NHS incubation and removal of bacteria) showed marked reduction of C5b-9 deposition on CHO cells and less hemolysis of red blood cells, arguing for a depletion of C5b-9 from the serum, probably due to binding to bacteria (Fig. S3). On a preliminary microscopy experiment, we stained C5b-9 at the surface of bacteria following serum incubation and observed foci of agglomerated signal on the regions immediately flanking the septum (data not shown). These results point towards a possible role of MAC assembly and the release of EVs but more studies are required to draw conclusions.

During its progression from the nasopharynx to other niches of the human host, *S. pneumoniae* must also adapt to changes in temperature. It has been shown that during pneumococcal spread, the temperature can range from 30 to 32 °C in the anterior nasopharynx, 37 °C in deeper tissues as the lungs, bloodstream, and central nervous system and up to 40 °C in feverish episodes [65]. Our data show that EVs production by *S. pneumoniae* D39 is sensitive to changes in temperature and it is enhanced at 37 °C. On the other hand, our results highlight that a pH shift negatively affects Sp-EVs production: exposure to either basic or acidic environments slows down pneumococcal EV formation (Fig. 2). The effect of high temperatures on bacterial EVs formation was also observed in *Staphylococcus aureus*, where not only more EVs were produced but also the cargo was altered [66]. Interestingly, one of the proteins enriched in the Sp-EVs proteome is glutamate dehydrogenase (GdhA), a protein recently shown to play crucial roles in pneumococcal thermoadaptation as well as for nutrient metabolism and virulence [65]. The presence of GdhA in EVs suggests a possible redistribution of key metabolic enzymes under certain environmental conditions. The enrichment of ribosomal proteins and RNA-related pathways in pneumococcal EVs likely reflects partial, non-selective encapsulation of cytoplasmic material during vesicle biogenesis.

Proteomic analysis also revealed that Sp-EVs are particularly enriched in proteins associated with carbon metabolism. The presence of so many essential metabolic enzymes in EVs is a remarkable and unprecedent observation. Rather than functioning as classical metabolic enzymes extracellularly, EV-associated glycolytic proteins likely act as effectors or signals reflecting the metabolic state of the cell and modulating host interactions or neighboring bacteria in a nutrient-dependent manner. Compelled by the presence of pneumolysin in the vesicles, in our previous study we investigated the hemolytic capacity of Sp-EVs but could not observe lysis at the used EVs-concentration [37]. The question remains if other enzymes in Sp-EVs have catalytic activity. Gram-negative bacteria-derived outer membrane vesicles (OMVs) have shown bactericidal activity due to the presence of one amidase in the vesicles [67]. EVs could be taken as *metabolic satellites*, protecting, distributing and delivering functional enzymes across cells and organisms.

Intriguingly, a moderate glucose concentration (10 mM) promoted Sp-EVs production (Fig. 4). *S. pneumoniae* relies on glucose as the primary carbon source for survival and growth [68]. Glucose is the prevailing sugar in blood and inflamed tissues and it is known to regulate the expression of virulence factors such as cell wall associated proteins [20]. Therefore, Sp-EVs production might be upregulated in favorable conditions, where glucose is present at moderate level (~ 10 mM), sufficient to support high glycolytic flux and activate regulatory systems to prioritize glucose consumption [57, 69]. Under variable glucose conditions, such as those encountered during early colonization or in certain tissue niches, the increase in EV production might serve as signalling to neighbouring cells or even as local nutrient scavenging systems. More studies on the cargo of Sp-EVs under glucose growing conditions are needed.

Finally, we demonstrate that Sp-EVs influence both intra- and interspecies biofilm formation and planktonic cells viability, suggesting that these vesicles play a role in intercellular communication (Fig. 5). It has already been reported that OMVs produced by *Helicobacter pylori*, a major etiologic factor in gastritis, act as a scaffold for cell-cell binding and increase biofilm formation on gastric epithelial cells [70]. In mixed *Streptococcus mutans - Candida albicans* biofilms, *S. mutans* produces EVs containing a factor that contributes to *C. albicans* biofilm formation [71]. We show that Sp-EVs enhance biofilm formation not only in *S. pneumoniae* but also in a different species, *S. pyogenes*, which can colonize the upper respiratory tract. However, Sp-EVs isolated under glucose-rich conditions promoted *S. pneumoniae* biofilm formation, whereas Sp-EVs from glucose-poor conditions increased *S. pyogenes* biofilm. This divergent effect on the two species may be linked to the Sp-EVs cargo, which could differentially influence biofilm development. Sp-EVs may play a major role in the transport of extracellular polymeric substance (EPS) components (such as extracellular polysaccharides, proteins and extracellular DNA), as well as nucleic acids, lipids and proteins [72], which exert a distinct effect on biofilm formation of two bacterial species. An increase in biofilm mass, following bacteria incubation with Sp-EVs, may indicate a shift towards a more stable state, which potentially increases the persistence of the pathogen in the host. This interspecies effect of EVs has been shown before. *Escherichia coli* EVs were found to inhibit group A *Streptococcus pyogenes* (GAS) peptidoglycan remodeling, inducing cell division defects and ultimately growth inhibition. Moreover, altered expression of many virulence genes has been reported in bacteria treated with *E. coli* EVs [73]. Although the mechanism remains unknown, Sp-EVs appear to serve as a platform for bacterial communication, likely delivering essential factors that promote biofilm development.

## Conclusions

Taken together, our study supports that EV formation is not solely a byproduct of bacterial physiology, but a regulated process closely linked to the metabolic and environmental status of the cell. By exploring how environmental conditions and nutrient availability shape pneumococcal EV production and how these vesicles contribute to biofilm formation and interspecies interactions, our study contributes to the understanding of how *S. pneumoniae* applies complex strategies to persist and spread, with potential future applications in infection control and therapeutic strategies. EVs are undoubtedly great delivery systems, but their active role on shaping bacterial metabolism deserves further attention. In the future, the intricate regulation of Sp-EVs formation should be dissected and their role as metabolic decoys explored.

## Supplementary Information

Supplementary Material 1.

## Data Availability

The datasets used and/or analysed during the current study are available from the corresponding author on reasonable request.

## Abbreviations

ATP: Adenosine triphosphate
CFU: Colony forming unit
CHO: Chinese hamster ovary
DAPI − 4′,6: Diamidino-2-phenylindole
DIC: Differential interference contrast
EV: Extracellular vesicle
GAS: Group A streptococcus
KEGG: Kyoto Encyclopedia of Genes and Genomes
MAC: Membrane attack complex
NA: Numerical aperture
NHS: Normal human serum
NTA: Nanoparticle tracking analysis
OD: Optical density
OMV: Outer membrane vesicle
PBST: Phosphate buffered saline-Tween
PFA: Paraformaldehyde
PPI: Protein-protein interaction
RT: Room temperature
SR-SIM: Super-Resolution Structured Illumination Microscopy
THY: Todd-Hewitt broth supplemented with yeast extract
WGA: Wheat germ agglutinin

## References

[1] Mao J, Blanchard AE, Lu T. Slow and steady wins the race: A bacterial exploitative competition strategy in fluctuating environments. ACS Synth Biol. 2015;4:240–8. 10.1021/sb4002008. 24635143 10.1021/sb4002008

[2] Shimizu K. Regulation Systems of Bacteria such as Escherichia coli in Response to Nutrient Limitation and Environmental Stresses. Metabolites. 2013;4:1–35. 10.3390/metabo4010001. 24958385 10.3390/metabo4010001 PMC4018673

[3] Stanley S, Wang X, Liu Q, Kwon YY, Frey AM, Hicks ND, et al. Ongoing evolution of the Mycobacterium tuberculosis lactate dehydrogenase reveals the pleiotropic effects of bacterial adaption to host pressure. PLoS Pathog. 2024;20. 10.1371/journal.ppat.1012050. 10.1371/journal.ppat.1012050 PMC10931510 38422159

[4] Cooper GA, Liu M, Peña J, West SA. The evolution of mechanisms to produce phenotypic heterogeneity in microorganisms. Nat Commun. 2022;13. 10.1038/s41467-021-27902-4. 10.1038/s41467-021-27902-4 PMC8789899 35078994

[5] La Rosa R, Johansen HK, Molina S. Convergent metabolic specialization through distinct evolutionary paths in Pseudomonas aeruginosa. mBio. 2018;9. 10.1128/mBio.00269-18. 10.1128/mBio.00269-18 PMC5893872 29636437

[6] Moreno-Gámez S. How bacteria navigate varying environments. Science. 2022;378. 10.1126/science.adf4444. 10.1126/science.adf4444 36423298

[7] Moreno-Gamez S, Hilla AL, Rosenbloom DIS, Petrov DA, Nowak MA, Pennings PS. Imperfect drug penetration leads to spatial monotherapy and rapid evolution of multidrug resistance. Proc Natl Acad Sci U S A. 2015;112. 10.1073/pnas.1424184112. 10.1073/pnas.1424184112 PMC4460514 26038564

[8] Ekkers DM, Tusso S, Moreno-Gamez S, Rillo MC, Kuipers OP, Van Doorn GS. Trade-Offs Predicted by Metabolic Network Structure Give Rise to Evolutionary Specialization and Phenotypic Diversification. Mol Biol Evol. 2022;39. 10.1093/molbev/msac124. 10.1093/molbev/msac124 PMC9206417 35679426

[9] Atolia E, Cesar S, Arjes HA, Rajendram M, Shi H, Knapp BD et al. Environmental and physiological factors affecting high-throughput measurements of bacterial growth. mBio. 2020;11. 10.1128/mBio.01378-20. 10.1128/mBio.01378-20 PMC7587430 33082255

[10] Lund PA, De Biase D, Liran O, Scheler O, Mira NP, Cetecioglu Z, et al. Understanding How Microorganisms Respond to Acid pH Is Central to Their Control and Successful Exploitation. Front Microbiol. 2020;11. 10.3389/fmicb.2020.556140. 10.3389/fmicb.2020.556140 PMC7553086 33117305

[11] Ratzke C, Gore J. Modifying and reacting to the environmental pH can drive bacterial interactions. PLoS Biol. 2018;16. 10.1371/journal.pbio.2004248. 10.1371/journal.pbio.2004248 PMC5868856 29538378

[12] Vilhena C, Kaganovitch E, Shin JY, Gruenberger A, Behr S, Kristoficova I, et al. A single-cell view of the BtsSR/YpdAB pyruvate sensing network in Escherichia coli and its biological relevance. J Bacteriol. 2018;200:e00536–17. 29038258 10.1128/JB.00536-17 PMC5717152

[13] Cornforth DM, Foster KR. Competition sensing: the social side of bacterial stress responses. Nat Rev Microbiol. 2013;11:285–93. 10.1038/nrmicro2977. 23456045 10.1038/nrmicro2977

[14] Olive AJ, Sassetti CM. Metabolic crosstalk between host and pathogen: Sensing, adapting and competing. Nat Rev Microbiol. 2016;14:221–34. 10.1038/nrmicro.2016.12. 26949049 10.1038/nrmicro.2016.12

[15] Fang FC, Frawley ER, Tapscott T, Vázquez-Torres A. Bacterial Stress Responses during Host Infection. Cell Host Microbe. 2016. 10.1016/j.chom.2016.07.009. 20. 27512901 10.1016/j.chom.2016.07.009 PMC4985009

[16] Barber MF, Fitzgerald JR. Mechanisms of host adaptation by bacterial pathogens. FEMS Microbiol Rev. 2024;48. 10.1093/femsre/fuae019. 10.1093/femsre/fuae019 PMC11308195 39003250

[17] Grant LR, Begier E, Theilacker C, Barry R, Hall-Murray C, Yan Q, et al. Multicountry Review of Streptococcus pneumoniae Serotype Distribution among Adults with Community-Acquired Pneumonia. J Infect Dis. 2024;229. 10.1093/infdis/jiad379. 10.1093/infdis/jiad379 PMC10786249 37665210

[18] Thadchanamoorthy V, Dayasiri K. Invasive Streptococcus Pneumoniae Septicemia Complicated with Hemolytic Uremic Syndrome and Meningitis. Cureus. 2020;12:12–6. 10.7759/cureus.10644. 10.7759/cureus.10644 PMC7586376 33133814

[19] Marion C, Stewart JM, Tazi MF, Burnaugh AM, Linke CM, Woodiga SA, et al. Streptococcus pneumoniae can utilize multiple sources of hyaluronic acid for growth. Infect Immun. 2012;80. 10.1128/IAI.05756-11. 10.1128/IAI.05756-11 PMC3318431 22311922

[20] Paixão L, Caldas J, Kloosterman TG, Kuipers OP, Vinga S, Neves AR. Transcriptional and metabolic effects of glucose on Streptococcus pneumoniae sugar metabolism. Front Microbiol. 2015. 10.3389/fmicb.2015.01041. 6 OCT. 26500614 10.3389/fmicb.2015.01041 PMC4595796

[21] Hernandez-Morfa M, Olivero NB, Zappia VE, Piñas GE, Reinoso-Vizcaino NM, Cian MB, et al. The oxidative stress response of Streptococcus pneumoniae: its contribution to both extracellular and intracellular survival. Front Microbiol. 2023;14. 10.3389/fmicb.2023.1269843. 10.3389/fmicb.2023.1269843 PMC10543277 37789846

[22] Fang Y, Wang Z, Liu X, Tyler BM. Biogenesis and Biological Functions of Extracellular Vesicles in Cellular and Organismal Communication With Microbes. Front Microbiol. 2022;13. 10.3389/fmicb.2022.817844. 10.3389/fmicb.2022.817844 PMC8895202 35250933

[23] Ñahui Palomino RA, Vanpouille C, Costantini PE, Margolis L. Microbiota–host communications: Bacterial extracellular vesicles as a common language. PLoS Pathog. 2021;17. 10.1371/journal.ppat.1009508. 10.1371/journal.ppat.1009508 PMC8118305 33984071

[24] Urabe F, Kosaka N, Ito K, Kimura T, Egawa S, Ochiya T. Extracellular vesicles as biomarkers and therapeutic targets for cancer. Am J Physiol Cell Physiol. 2020;318. 10.1152/ajpcell.00280.2019. 10.1152/ajpcell.00280.2019 31693397

[25] Kim JH, Lee J, Park J, Gho YS. Gram-negative and Gram-positive bacterial extracellular vesicles. Semin Cell Dev Biol. 2015;40:97–104. 10.1016/j.semcdb.2015.02.006. 25704309 10.1016/j.semcdb.2015.02.006

[26] Zaborowski MP, Balaj L, Breakefield XO, Lai CP. Extracellular Vesicles: Composition, Biological Relevance, and Methods of Study. Bioscience. 2015;65. 10.1093/biosci/biv084. 10.1093/biosci/biv084 PMC4776721 26955082

[27] Kumar MA, Baba SK, Sadida HQ, Marzooqi S, Al, Jerobin J, Altemani FH, et al. Extracellular vesicles as tools and targets in therapy for diseases. Signal Transduct Target Therapy. 2024;9. 10.1038/s41392-024-01735-1. 10.1038/s41392-024-01735-1 PMC10838959 38311623

[28] Effah CY, Ding X, Drokow EK, Li X, Tong R, Sun T. Bacteria-derived extracellular vesicles: endogenous roles, therapeutic potentials and their biomimetics for the treatment and prevention of sepsis. Front Immunol. 2024;15. 10.3389/fimmu.2024.1296061. 10.3389/fimmu.2024.1296061 PMC10899385 38420121

[29] Brown L, Wolf JM, Prados-Rosales R, Casadevall A. Through the wall: Extracellular vesicles in Gram-positive bacteria, mycobacteria and fungi. Nat Rev Microbiol. 2015;13:620–30. 10.1038/nrmicro3480. 26324094 10.1038/nrmicro3480 PMC4860279

[30] Saad MG, Beyenal H, Dong WJ. Dual roles of the conditional extracellular vesicles derived from Pseudomonas aeruginosa biofilms: Promoting and inhibiting bacterial biofilm growth. Biofilm. 2024. 10.1016/j.bioflm.2024.100183. 7. 38380422 10.1016/j.bioflm.2024.100183 PMC10876606

[31] Halder LD, Jo EAH, Hasan MZ, Ferreira-Gomes M, Krüger T, Westermann M, et al. Immune modulation by complement receptor 3-dependent human monocyte TGF-β1-transporting vesicles. Nat Commun. 2020;2331. 10.1038/s41467-020-16241-5. 10.1038/s41467-020-16241-5 PMC7214408 32393780

[32] Yerneni SS, Werner S, Azambuja JH, Ludwig N, Eutsey R, Lucas PC, et al. Pneumococcal extracellular vesicles modulate host immunity. mBio. 2021;12. 10.1128/mBio.01657-21. 10.1128/mBio.01657-21 PMC8406339 34253061

[33] Mehanny M, Boese A, Bornamehr B, Hoppstädter J, Presser V, Kiemer AK, et al. Spray-dried pneumococcal membrane vesicles are promising candidates for pulmonary immunization. Int J Pharm. 2022;621:121794. 10.1016/j.ijpharm.2022.121794. 35525468 10.1016/j.ijpharm.2022.121794

[34] Mehanny M, Kroniger T, Koch M, Hoppstädter J, Becher D, Kiemer AK, et al. Yields and Immunomodulatory Effects of Pneumococcal Membrane Vesicles Differ with the Bacterial Growth Phase. Adv Healthc Mater. 2022;11:1–16. 10.1002/adhm.202101151. 10.1002/adhm.202101151 PMC11469037 34724354

[35] Parveen S, Subramanian K. Emerging Roles of Extracellular Vesicles in Pneumococcal Infections: Immunomodulators to Potential Novel Vaccine Candidates. Front Cell Infect Microbiol. 2022;12:1–8. 10.3389/fcimb.2022.836070. 10.3389/fcimb.2022.836070 PMC8882830 35237534

[36] Olaya-Abril A, Prados-Rosales R, González-Reyes JA, Casadevall A, Pirofski LA, Rodríguez-Ortega MJ. Extracellular vesicles from different pneumococcal serotypes are internalized by macrophages and induce host immune responses. Pathogens. 2021;10. 10.3390/pathogens10121530. 10.3390/pathogens10121530 PMC8708143 34959485

[37] Battista M, Hoffmann B, Bachelot Y, Zimmermann L, Teuber L, Jost A, et al. The role of pneumococcal extracellular vesicles on the pathophysiology of the kidney disease hemolytic uremic syndrome. mSphere. 2023. 10.1128/msphere.00142-23. 37358300 10.1128/msphere.00142-23 PMC10449520

[38] Lanie JA, Ng WL, Kazmierczak KM, Andrzejewski TM, Davidsen TM, Wayne KJ, et al. Genome sequence of Avery’s virulent serotype 2 strain D39 of Streptococcus pneumoniae and comparison with that of unencapsulated laboratory strain R6. J Bacteriol. 2007;189:38–51. 10.1128/JB.01148-06. 17041037 10.1128/JB.01148-06 PMC1797212

[39] Ferretti JJ, McShan WM, Ajdic D, Savic DJ, Savic G, Lyon K, et al. Complete genome sequence of an M1 strain of streptococcus pyogenes. Proc Natl Acad Sci U S A. 2001;98. 10.1073/pnas.071559398. 10.1073/pnas.071559398 PMC31890 11296296

[40] Schindelin J, Arganda-Carreras I, Frise E, Kaynig V, Longair M, Pietzsch T, et al. Fiji: an open-source platform for biological-image analysis. Nat Methods. 2012;9:676–82. 10.1038/nmeth.2019. 22743772 10.1038/nmeth.2019 PMC3855844

[41] Camacho C, Coulouris G, Avagyan V, Ma N, Papadopoulos J, Bealer K, et al. BLAST+: Architecture and applications. BMC Bioinformatics. 2009;10. 10.1186/1471-2105-10-421. 10.1186/1471-2105-10-421 PMC2803857 20003500

[42] Szklarczyk D, Kirsch R, Koutrouli M, Nastou K, Mehryary F, Hachilif R, et al. The STRING database in 2023: protein-protein association networks and functional enrichment analyses for any sequenced genome of interest. Nucleic Acids Res. 2023;51:1D. 10.1093/nar/gkac1000. 36370105 10.1093/nar/gkac1000 PMC9825434

[43] Shannon P, Markiel A, Ozier O, Baliga NS, Wang JT, Ramage D, et al. Cytoscape: A software Environment for integrated models of biomolecular interaction networks. Genome Res. 2003;13. 10.1101/gr.1239303. 10.1101/gr.1239303 PMC403769 14597658

[44] O’Toole GA. Microtiter dish Biofilm formation assay. J Visualized Experiments. 2010;10–1. 10.3791/2437. 10.3791/2437 PMC3182663 21307833

[45] Ledger EVK, Edwards AM. Host-induced cell wall remodeling impairs opsonophagocytosis of Staphylococcus aureus by neutrophils. mBio. 2024;15. 10.1128/mbio.01643-24. 10.1128/mbio.01643-24 PMC11323798 39041819

[46] Ledger EVK, Massey RC. PBP4 is required for serum-induced cell wall thickening and antibiotic tolerance in *Staphylococcus aureus*. Antimicrob Agents Chemother. 2024;68. 10.1128/aac.00961-24. 10.1128/aac.00961-24 PMC11539222 39431816

[47] Gnopo YMD, Misra A, Hsu HL, DeLisa MP, Daniel S, Putnam D. Induced fusion and aggregation of bacterial outer membrane vesicles: Experimental and theoretical analysis. J Colloid Interface Sci. 2020;578:522–32. 10.1016/j.jcis.2020.04.068. 32540551 10.1016/j.jcis.2020.04.068 PMC7487024

[48] McMillan HM, Kuehn MJ. The extracellular vesicle generation paradox: a bacterial point of view. EMBO J. 2021;40. 10.15252/embj.2021108174. 10.15252/embj.2021108174 PMC8561641 34636061

[49] Szklarczyk D, Franceschini A, Kuhn M, Simonovic M, Roth A, Minguez P, et al. The STRING database in 2011: Functional interaction networks of proteins, globally integrated and scored. Nucleic Acids Res. 2011;39(SUPPL 1). 10.1093/nar/gkq973. 10.1093/nar/gkq973 PMC3013807 21045058

[50] Hong J, Dauros-Singorenko P, Whitcombe A, Payne L, Blenkiron C, Phillips A, et al. Analysis of the Escherichia coli extracellular vesicle proteome identifies markers of purity and culture conditions. J Extracell Vesicles. 2019;8. 10.1080/20013078.2019.1632099. 10.1080/20013078.2019.1632099 PMC6598517 31275533

[51] Plavec TV, Žagar Soderžnik K, Della Pelle G, Zupančič Š, Vidmar R, Berlec A. Incorporation of recombinant proteins into extracellular vesicles by Lactococcus cremoris. Sci Rep. 2025;15:1768. 10.1038/s41598-025-86492-z. 39815011 10.1038/s41598-025-86492-z PMC11736121

[52] Hirayama S, Domon H, Hiyoshi T, Isono T, Tamura H, Sasagawa K, et al. Triosephosphate isomerase of Streptococcus pneumoniae is released extracellularly by autolysis and binds to host plasminogen to promote its activation. FEBS Open Bio. 2022;12. 10.1002/2211-5463.13396. 10.1002/2211-5463.13396 PMC9157410 35298875

[53] Bergmann S, Rohde M, Chhatwal GS, Hammerschmidt S. α-Enolase of Streptococcus pneumoniae is a plasmin(ogen)-binding protein displayed on the bacterial cell surface. Mol Microbiol. 2001;40. 10.1046/j.1365-2958.2001.02448.x. 10.1046/j.1365-2958.2001.02448.x 11442827

[54] Bergmann S, Rohde M, Hammerschmidt S. Glyceraldehyde-3-Phosphate Dehydrogenase of Streptococcus pneumoniae Is A Surface-Displayed Plasminogen-Binding Protein. Infect Immun. 2004. 10.1128/IAI.72.4.2416-2419.2004. 72. 15039372 10.1128/IAI.72.4.2416-2419.2004 PMC375162

[55] Carvalho SM, Kloosterman TG, Kuipers OP, Neves AR. CcpA ensures optimal metabolic fitness of streptococcus pneumoniae. PLoS ONE. 2011;6. 10.1371/journal.pone.0026707. 10.1371/journal.pone.0026707 PMC3198803 22039538

[56] Echlin H, Frank M, Rock C, Rosch JW. Role of the pyruvate metabolic network on carbohydrate metabolism and virulence in Streptococcus pneumoniae. Mol Microbiol. 2020;114. 10.1111/mmi.14557. 10.1111/mmi.14557 PMC8538403 32495474

[57] Werren JP, Mostacci N, Gjuroski I, Holivololona L, Troxler LJ, Hathaway LJ, et al. Carbon source–dependent capsule thickness regulation in Streptococcus pneumoniae. Front Cell Infect Microbiol. 2023;13. 10.3389/fcimb.2023.1279119. 10.3389/fcimb.2023.1279119 PMC10716237 38094742

[58] Deng Y, Beahm DR, Ionov S, Sarpeshkar R. Measuring and modeling energy and power consumption in living microbial cells with a synthetic ATP reporter. BMC Biol. 2021;19. 10.1186/s12915-021-01023-2. 10.1186/s12915-021-01023-2 PMC8130387 34001118

[59] Pandey S, Blache A, Achouak W. Insights into Bacterial Extracellular Vesicle Biogenesis, Functions, and Implications in Plant–Microbe Interactions. Microorganisms. 2024;12. 10.3390/microorganisms12030532. 10.3390/microorganisms12030532 PMC10975234 38543583

[60] Chen N, Li Y, Liang X, Qin K, Zhang Y, Wang J, et al. Bacterial extracellular vesicle: A non-negligible component in biofilm life cycle and challenges in biofilm treatments. Biofilm. 2024;8. 10.1016/j.bioflm.2024.100216. 10.1016/j.bioflm.2024.100216 PMC11341940 39184814

[61] Bessen DE, Smeesters PR, Beall BW. Molecular Epidemiology, Ecology, and Evolution of Group A Streptococci. Microbiol Spectr. 2018;6. 10.1128/microbiolspec.cpp3-0009-2018. 30191802 10.1128/microbiolspec.cpp3-0009-2018 PMC11633622

[62] Cook CC, Lasarre LC, Federle BJ. Interspecies communication among commensal and pathogenic streptococci. mBio. 2013;4:382–95. 10.1128/mBio.00382. 10.1128/mBio.00382-13 PMC3735184 23882015

[63] Gómez-Mejia A, Gámez G, Hammerschmidt S. Streptococcus pneumoniae two-component regulatory systems: The interplay of the pneumococcus with its environment. Int J Med Microbiol. 2018;308:722–37. 10.1016/j.ijmm.2017.11.012. 29221986 10.1016/j.ijmm.2017.11.012

[64] Pathak A, Bergstrand J, Sender V, Spelmink L, Aschtgen MS, Muschiol S, et al. Factor H binding proteins protect division septa on encapsulated Streptococcus pneumoniae against complement C3b deposition and amplification. Nat Commun. 2018;9:1–16. 10.1038/s41467-018-05494-w. 30139996 10.1038/s41467-018-05494-w PMC6107515

[65] Gazioglu O, Kareem BO, Afzal M, Shafeeq S, Kuipers OP, Ulijasz AT, Andrew PW, Yesilkaya H. Glutamate dehydrogenase (GdhA) of *Streptococcus pneumoniae* is required for high temperature adaptation. Infect Immun. 2021;89(12):e0040021. Epub 2021 Sep 7. PMID: 34491792; PMCID: PMC8594611. 10.1128/IAI.00400-21. 10.1128/IAI.00400-21 PMC8594611 34491792

[66] Briaud P, Frey A, Marino EC, Bastock RA, Zielinski RE, Wiemels RE et al. Temperature Influences the Composition and Cytotoxicity of Extracellular Vesicles in Staphylococcus aureus. mSphere. 2021;6. 10.1128/msphere.00676-21. 10.1128/mSphere.00676-21 PMC8510519 34612674

[67] Chen Y-C, Kalawong R, Toyofuku M, Eberl L. The role of peptidoglycan hydrolases in the formation and toxicity of Pseudomonas aeruginosa membrane vesicles. microLife. 2022;3. 10.1093/femsml/uqac009. 10.1093/femsml/uqac009 PMC10117874 37229443

[68] Leonard A, Lalk M. Infection and metabolism – Streptococcus pneumoniae metabolism facing the host environment. Cytokine. 2018;112:75–86. 10.1016/j.cyto.2018.07.021. 30077545 10.1016/j.cyto.2018.07.021

[69] Iyer R, Baliga NS, Camilli A. Catabolite control protein A (CcpA) contributes to virulence and regulation of sugar metabolism in Streptococcus pneumoniae. J Bacteriol. 2005;187:8340–9. 10.1128/JB.187.24.8340-8349.2005. 16321938 10.1128/JB.187.24.8340-8349.2005 PMC1317011

[70] Yonezawa H, Osaki T, Kurata S, Fukuda M, Kawakami H, Ochiai K, et al. Outer membrane vesicles of helicobacter pylori TK1402 are involved in biofilm formation. BMC Microbiol. 2009;9. 10.1186/1471-2180-9-197. 10.1186/1471-2180-9-197 PMC2749055 19751530

[71] Wu R, Tao Y, Cao Y, Zhou Y, Lin H. Streptococcus mutans Membrane Vesicles Harboring Glucosyltransferases Augment Candida albicans Biofilm Development. Front Microbiol. 2020;11. 10.3389/fmicb.2020.581184. 10.3389/fmicb.2020.581184 PMC7517897 33042098

[72] Jeong GJ, Khan F, Tabassum N, Cho KJ, Kim YM. Bacterial extracellular vesicles: Modulation of biofilm and virulence properties. Acta Biomater. 2024;178. 10.1016/j.actbio.2024.02.029. 10.1016/j.actbio.2024.02.029 38417645

[73] Kawagishi Y, Murase K, Grebenshchikova A, Iibushi J, Ma C, Kimeu TM, et al. Bacterial extracellular vesicles target different bacterial species, impairing cell division and diminishing their pathogenicity. Proc Natl Acad Sci U S A. 2025;122. 10.1073/pnas.2416652122. 10.1073/pnas.2416652122 PMC12067206 40299696

