# Supplementary material for: Environmental cues shape extracellular vesicles biogenesis and function in Streptococcus pneumoniae
Source: BMC Microbiol. 2026 Mar 4;26:320. doi: 10.1186/s12866-026-04862-7 (PMC13064100; doi:10.1186/s12866-026-04862-7)
Supplement: Supplementary file 1 — Supplementary Material 1. [file 12866_2026_4862_MOESM1_ESM.pdf]

Supplementary Material

Environmental cues shape extracellular vesicles biogenesis and function in *Streptococcus pneumoniae*

Miriana Battista[1], Yann Bachelot[2,3], Teresa Franke[1], Christoph Saffer[2], Lioba Zimmermann[4], Laura Teuber[4], Marc Thilo Figge[2,5], Cláudia Vilhena[#1,4]

[1]Friedrich-Alexander-Universität Erlangen-Nürnberg, Department of Biology, Bacterial Interface Dynamics lab, Erlangen, Germany
[2]HKI-Center for Systems Biology of Infection, Leibniz-Institute for Natural Product Research and Infection Biology, Department of Applied Systems Biology, Jena, Germany
[3]Friedrich-Schiller-University, Jena, Germany
[4]Leibniz Institute for Natural Product Research and Infection Biology, Department of Infection Biology, Jena, Germany
[5]Friedrich-Schiller-University, Institute of Microbiology, Jena, Germany

Corresponding author:

Dr. Cláudia Vilhena

https://orcid.org/0000-0003-2884-8970

Junior Research Group Leader- *Bacterial Interface Dynamics lab*
Department of Biology, Chair of Pharmaceutical Biology
Friedrich-Alexander University Erlangen Nürnberg
Staudtstraße 5, 91058 Erlangen

Running Title: Pneumococcal EVs as metabolic satellites

Keywords: metabolism, biofilm, carbon source, vesicles, biogenesis, intercellular communication

Supplementary Methods

Chinese Hamster Ovary Cells (CHO cells)

CHO cells were used for complement deposition studies. CHO cells were chosen because they do not spontaneously activate or express any regulators of human complement and therefore should not resist complement deposition induced by human serum[1]. The cells were obtained from the Leibniz-Institute German Collection of Microorganisms and Cell Cultures (DSMZ). Cells were cultured in Dulbecco's modified Eagle's medium (DMEM) supplemented with 10% Fetal Bovine Serum (FBS), 2 mM ultraglutamine 1, 100 units/ml Penicillin, 0.1 mg/ml Streptomycin and 2.5 µg/ml Amphotericin B solution at 37 °C and 5% CO^2 in either 25 cm^2 or 75 cm^2 flasks (Cellstar®). Cells were detached from the flask by treatment with Trypsin/Ethylenediaminetetraacetic acid (EDTA) Solution (0.25% / 0.02% w/v in Phosphate buffered saline (PBS) w/o Ca2+ and Mg2+) for approximately 10 minutes at 37 °C. The reaction was stopped by adding pre-warmed full supplemented DMEM, which was subsequently removed by centrifugation at 90 x g for 10 minutes at room temperature (RT). After resuspension of the cell pellet in fresh DMEM, cells were counted on a cell-counter (CASY) and subsequently transferred to a new flask.

Flow cytometry for C5b-9 deposition on bacteria

To assess C5b-9 deposition on bacteria cells, flow cytometry was performed. *S. pneumoniae* D39 strain was incubated with NHS as explained in the Material and Methods section on the main text. As controls for flow cytometry experiments, additional samples of *S. pneumoniae* were incubated with THY medium. Between all the following steps centrifugations were performed at 7,200 x g for 3 minutes at 4 °C. Unspecific binding sites on the bacterial cell surfaces were blocked with 1% Bovine Serum Albumin (BSA) in DPBS for 45 minutes at RT. C5b-9 deposits were targeted by adding murine anti-C5b-9 primary antibody (1:1000) in 1%

BSA. Incubation followed for 1 hour at RT. After washing with PBS containing 0.5% Tween solution (PBST), bacteria were incubated with anti-mouse AlexaFluor®-488 secondary antibody (1:1000) in PBST for 1 hour at RT. After washing with PBST, the bacterial pellet was resuspended in DPBS. The fluorescence intensity of the AlexaFluor®-488 antibody was measured for 20,000-50,000 events with the BD Accuri™ C6 Plus Flow Cytometer. The data were analyzed using the software FlowJo. Unstained samples, which were only incubated in THY, were kept at 4 °C after the BSA incubation until the flow cytometry measurements.

Flow cytometry for C5b-9 deposition on CHO cells

Confluent CHO cells were detached from their flask and their concentration ascertained. Approximately 500,000 cells were pelleted, resuspended in 400 µl serum and incubated for 30 minutes at 37 °C. All subsequent steps of the immunostaining protocol were performed as described above with some changes. The centrifugations between steps were performed at 4,800 x g for 2 minutes, at RT. Bacteria were incubated with murine anti-C5b-9 primary antibody (1:50) in 1% BSA and the anti-mouse AlexaFluor®-488 secondary antibody (1:500) in PBST. During the final step, the CHO pellet was resuspended in 100 µl DPBS and 10,000 events were recorded with the Flow Cytometer.

Hemolysis

Hemolysis assay was performed to assess the serum activity of NHS. The assay was carried out using rabbit erythrocytes to avoid inactivation of the complement derived from human serum. In a round-bottom polystyrene 96-well plate (Nunclon™) 100 µl serum were added to 100 µl rabbit erythrocytes (from a 50% stock in DPBS) and incubated at 37 °C at 400 rpm for 30 minutes. THY medium and DPBS were used as negative controls. As a positive control double distilled water was added during the last 5 minutes of the incubation. As further controls 100 µl each of a D39 and HUSA growing suspension (approx. 2x107 CFUs) were

used. After centrifugation at 450 x g for 15 minutes at RT, 90 µl of the supernatants were transferred to a flat-bottom plate. The serum activity was assessed by measuring the absorbance of released hemoglobin in the supernatants at 540 nm with a plate reader (Tecan safire 2). The data were analyzed using the software GraphPad Prism 8.

Supplementary Figures and Figure Legends

Fig. S1

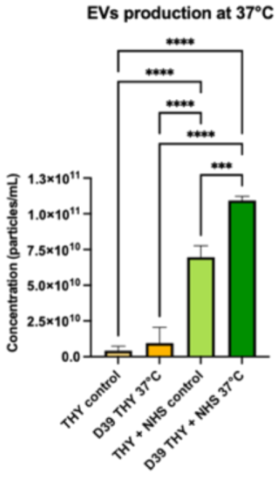

Fig. S1 Comparison of EVs production from bacteria and media controls. Graph representing EVs concentration (particles/mL). EVs were isolated from the supernatant of *S. pneumoniae* D39 grown until mid-logarithmic phase in THY at 37°C with or without NHS. Controls for EVs isolated from THY with or without NHS alone without bacteria were included. Mean ± SD (n=3). Ordinary one-way ANOVA test with Tukey's multiple comparisons test.

Fig. S2

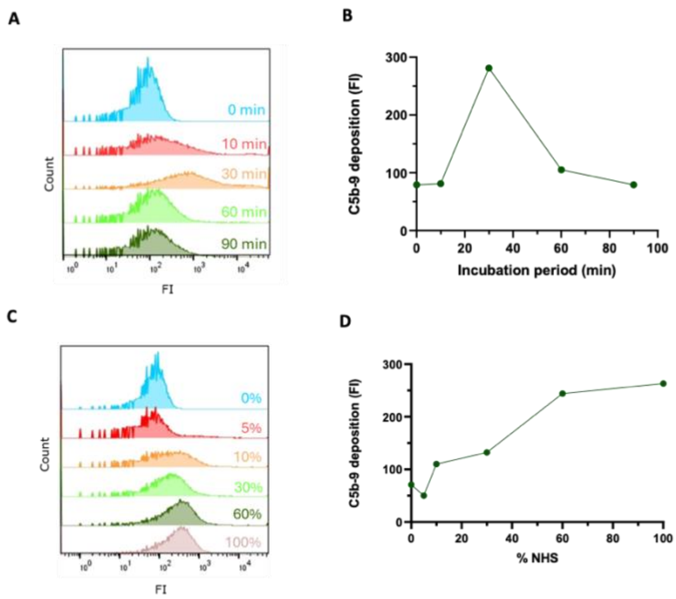

Fig. S2 Optimization of the incubation of *S. pneumoniae* with normal human serum (NHS) to assess C5b9-deposition. (**A**) Histograms showing the AF®- 488 fluorescent intensity (FI) for each incubation length. Incubation of D39 cells were performed for 10 min (red), 30 min (orange), 60 min (light green) and 90 min (dark green) with 20% NHS (diluted in THY). Pneumococci incubated in THY medium served as the 0 min control (blue). (**B**) Line graph representing the median fluorescent intensity (FI) expressed in AU of each incubation length. (**C**) Histograms showing the AF®-488 fluorescent intensity for each NHS concentration. Incubation of D39 cells were performed with 5% (red), 10% (orange), 30% (light green), 60% (dark green) and 100% (pink) NHS (diluted in THY) for 30 min. Pneumococci incubated in THY medium served as the 0% control (blue). (**D**) Line graph representing the median fluorescent intensity (FI) expressed in AU of each NHS concentration.

Fig. S3

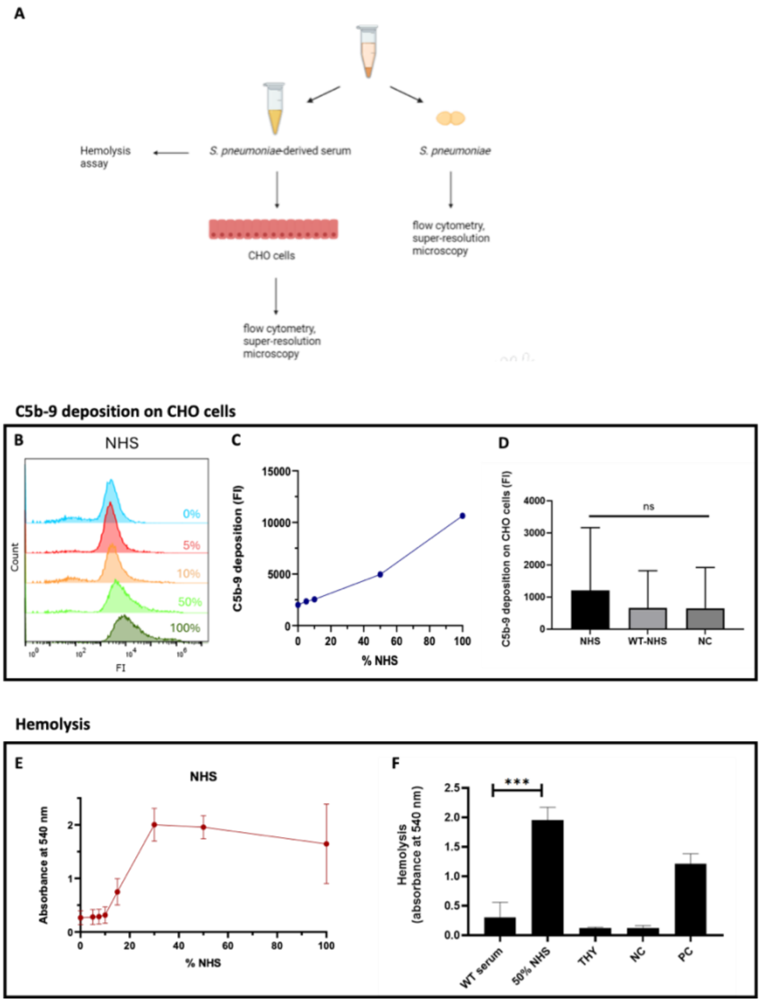

Fig. S3 C5b-9 deposition on CHO cells after NHS incubation and hemolytic activity of NHS. (**A**) Schematic representation of the experiments performed to assess C5b-9 deposition on CHO cells and NHS hemolytic activity. (**B**) Histograms showing the AF®-488 fluorescent intensity (FI) for each NHS concentration. CHO cells were incubated with 5% (red), 10% (orange), 50% (light green) and 100% NHS (dark green, diluted in DMEM) for 30 min. CHO cells incubated only in DMEM served as the 0% control (blue). (**C**) Line graph representing the median fluorescent intensity (FI) of each NHS concentration. (**D**) Bar graph representing the AF®-488 median fluorescent intensity (FI) for 50% NHS, *S. pneumoniae*-derived NHS (WT-NHS) and the negative control (CHO cells in DMEM) conditions. (**E**) Graph showing the hemolytic activity. The hemolytic activity of NHS used at 5%, 7.5%, 10%, 15%, 30%, 50% and 100% was examined. NHS (diluted in THY) was incubated with rabbit erythrocytes for 30 min

at 37 °C. Erythrocytes incubated in only THY served as the 0% control. (**F**) Bar graph showing the absorbance of the supernatants, measured at 540 nm, corresponding to the hemolytic activity of *S. pneumoniae*-derived NHS (WT-serum), 50% NHS, THY, DPBS (NC, negative control) and double distilled water (PC, positive control). Means ± SE. Ordinary one-way ANOVA test with Tukey’s multiple comparisons test.

References for this section:

(1) Liszewski, M. K.; Fang, C. J.; Atkinson, J. P. Inhibiting Complement Activation on Cells at the Step of C3 Cleavage. *Vaccine* 2008, *26* (SUPPL. 8). https://doi.org/10.1016/j.vaccine.2008.11.001.
